# Caregiver-child discrepancy in healthcare transition readiness and its associations

**DOI:** 10.1016/j.hctj.2025.100113

**Published:** 2025-07-26

**Authors:** Yunzhen Huang, Eugene Maung, Stephen R. Hooper, Chad Coltrane, Maria Díaz-González de Ferris

**Affiliations:** a Department of Health Sciences, School of Medicine, University of North Carolina at Chapel Hill, United States; b Edward Via College of Osteopathic Medicine – Carolinas Campus, United States; c Victory Junction Camp, United States; d Department of Pediatrics, School of Medicine, University of North Carolina at Chapel Hill, United States

**Keywords:** Caregiver-child discrepancy, Chronic health conditions, Pediatric anxiety, Health services utilization

## Abstract

**Purpose:**

To explore caregiver-child discrepancy in healthcare transition (HCT) readiness and its association with demographic variables, anxiety, and health services utilization in children and adolescents with chronic health conditions.

**Methods:**

This cross-sectional study surveyed 214 caregiver-child dyads recruited from a therapeutic camp in the Southeastern United States. Children and adolescents aged 7–17 years and their caregivers completed the STAR_x_ Questionnaire to assess HCT readiness. Additionally, children rated their anxiety using the PROMIS-Anxiety scale, and caregivers reported their child’s past-year health services utilization. Paired *t*-tests were used to examine the caregiver-child discrepancies in HCT readiness. Correlation analyses and linear regression were used to explore factors associated with caregiver-child discrepancies in HCT readiness.

**Results:**

No statistically significant discrepancies were identified at the full-scale and subscale levels of the STAR_x_ Questionnaire. However, single-item level analysis showed caregiver-child discrepancies in their perception of the child’s medication adherence and disease knowledge. Caregivers generally rated children’s HCT readiness higher than children did themselves, particularly in younger children and those diagnosed at a younger age. Higher caregiver ratings were correlated with greater child anxiety.

**Conclusion:**

This study revealed gaps in caregiver-child perceptions of the child’s HCT readiness. Addressing these gaps through collaborative communication, shared decision-making, and targeted interventions may improve the HCT process and outcomes. Additionally, this study showed that greater caregiver ratings were linked to younger age, younger age at diagnoses, and elevated child anxiety, calling for early, effective interventions for transition planning to mitigate differences in caregiver-child ratings and facilitate HCT.

## Highlights

• Caregivers generally rated children’s transition readiness higher than children.
• Children report missing medications more frequently than caregivers’ proxy reports.
• Higher caregiver ratings were correlated with younger child age and diagnosis age.
• Higher caregiver ratings were correlated with greater child anxiety.

## 1 Introduction

Healthcare transition (HCT) involves the process of shifting from pediatric- to adult-focused care for adolescents and young adults (AYAs) with chronic conditions, defined as individuals aged 10–24 years.^1^ It is of unique importance in the context of medical complexity, as the life expectancy for many chronic conditions in pediatric patients continues to improve.^2^, ^3^ Ensuring HCT readiness is vital to preventing loss to follow-up as well as promoting survival and quality of life among these patients.^4^, ^5^ A key component of the HCT process is the measurement of HCT readiness, which encompasses the AYAs’ capacity for self-management.^5^ It is recommended that HCT readiness assessment and preparation begin as early as age 12 and no later than age 17 to allow sufficient time to develop self-management skills.^6^ For children and adolescents with special health care needs, the preparation process may need to begin even earlier.^7^

Measurement of HCT readiness often involves surveys that assess both child and caregiver perspectives.^8^ Caregiver input is a vital metric, as it provides valuable insight from the individuals who play a major role in the management of the medically complex youth. However, studies have reported notable discrepancies between caregiver and child reports,^9^, ^10^ which can lead to inaccurate assessments of transition readiness, misaligned care plans, and reduced engagement in shared decision-making.^9^, ^11^ It is important to recognize and reconcile such discrepancies to determine appropriate interventions and referral pathways to ensure a smooth HCT.

Anxiety can significantly affect both self-perceptions and caregiver perceptions of HCT readiness. Anxiety is a common comorbidity in children and adolescents with chronic conditions^12^ and has been linked to poorer HCT readiness.^13^ In several qualitative studies, patients identified their anxiety as a barrier to successful HCT, which hindered their communication with the adult care team^14^ and their ability to prioritize disease-related healthcare needs.^15^

The current study aims to further investigate the caregiver-child discrepancy in HCT readiness by self- and caregiver-proxy reports and examine its associations with demographic factors, anxiety, and health services utilization. Given that self-management skills begin to develop during middle childhood (ages 6–11),^16^ the current study extends the recommended HCT preparation age range to include younger children and adolescents aged 7–17 years. The research questions (RQ) are: (1) What is the extent of discrepancy between child self-reports and caregiver-proxy reports regarding readiness for HCT? (2) What are the correlations between caregiver-child discrepancies in HCT readiness and demographic factors, child anxiety levels, and health services utilization in children and adolescents?

## 2 Methods

### 2.1 Participants, procedure, and ethics

The study was approved by the institutional review board of the University of North Carolina at Chapel Hill (IRB study # 17–1547). During the summer of 2018, recruitment emails containing the study information and the Qualtrics™ survey link were sent to caregivers of children and adolescents with chronic conditions who participated in a therapeutic camp Victory Junction. Located in Randleman, NC in the Southeastern United States, the camp serves children and adolescents with chronic medical conditions. In week-long programs, the therapeutic camp is staffed by nurses, physicians, and advanced medical providers, so campers can enjoy recreational activities (e.g., horseback riding, bowling, fishing, or swimming) in a medically safe environment. Caregiver-child dyads were eligible to participate if (a) both caregiver and child were proficient in English, (b) the child’s chronological age was between 7 and 17 years, and (c) the child was able to answer survey questions independently.

The Qualtrics™ survey included electronic consent and assent forms, as well as study measures for caregivers and children, respectively. At the beginning of the survey, caregivers were presented with an electronic consent form and were asked to provide consent for their own and their children’s participation by selecting two separate “I agree” responses. Upon providing consent, caregivers completed their portion of the survey. If they deemed it appropriate (based on their child’s age and cognition or ability to respond to the surveys), caregivers were asked to share the Qualtrics™ survey link with their children. Children were presented with an electronic assent form written in developmentally appropriate and simplified language, and could proceed to complete the survey only after selecting “I agree” to participate in the study.

### 2.2 Measures

#### 2.2.1 Demographic questionnaire

Caregivers were asked about their relationship to the child, level of education, employment status, as well as the child’s race, primary chronic condition, comorbid mood disorders, and health services utilization in the last 12 months (emergency room, hospitalizations, and length of stay). In the child version, children reported their age and sex.

#### 2.2.2 HCT readiness by STAR_x_ Questionnaire child and caregiver-proxy versions

The research version of the Self-Management and Transition to Adulthood with R_x_ = Treatment (STAR_x_) Questionnaire^8^ was used to assess the child’s HCT readiness. It has 13 items rated on a 5-point Likert scale (1 = “never”, “nothing”, or “very hard”, 5 = “always”, “a lot”, or “very easy”). The STAR_x_ consists of three subscale domains: disease knowledge, self-management, and provider communication. The four medication-related items of the scale include an additional “N/A” response to indicate that medication is not needed for the child’s care. The “N/A” responses were treated as missing values.

The STAR_x_ Questionnaire has two versions: the child version (STAR_x_-C)^8^ and the caregiver-proxy version (STAR_x_-P).^17^ Both the child (STAR_x_-C) and caregiver-proxy versions (STAR_x_-P) were used in this study. In this study, Cronbach’s alpha for the child version was.83 for the full scale, and 0.66, 0.64, and 0.72 for the disease knowledge, self-management, and provider communication subscales, respectively. The Cronbach’s alpha for the caregiver-proxy version was 0.86 for the full scale, and 0.66, 0.71, and 0.81 for the three subscales, respectively.

#### 2.2.3 Child anxiety by PROMIS-A Questionnaire

The Pediatric Patient-Reported Outcomes Measurement Information System-Anxiety Scale (PROMIS-A)^18^, ^19^ assesses the anxiety symptoms of children and adolescents in clinical settings. The child self-reported version was used in this study. The PROMIS-A has eight items rated on a 5-point Likert scale (0 = “never”, 4 = “almost always”). In this study, the Cronbach’s alpha for PROMIS-A was 0.90.

### 2.3 Statistical analysis

All statistical analyses were conducted in SPSS 28.^20^ Expectation maximization^21^ was used to impute the missing values resulting from the “N/A” responses in the STAR_x_ Questionnaire. For RQ1, paired *t*-tests were used to examine the pairwise differences between child- and caregiver proxy-reported HCT readiness at the full-scale level, subscale domains, and single-item level. The examination of single-item level discrepancies is clinically relevant because it may help clinicians to identify specific areas of caregiver and child misalignment, which can inform targeted interventions for communication improvement, shared decision-making, and HCT readiness. Bonferroni corrections for multiple comparisons in the given subscale were performed for paired *t*-tests at the single-item level by dividing the significance level (α =.05) by the number of items on the given subscale.^22^

For RQ2, the standardized difference score (SDS)^23^, ^24^ was used to measure the discrepancy between child and caregiver-proxy reports on HCT readiness. SDS was calculated by subtracting the *z*-score of the child’s self-reported rating from the corresponding *z*-score of the caregiver-proxy rating. A positive SDS indicates a higher caregiver report, and a negative SDS indicates a higher child report.

Pearson correlation analyses were conducted to identify statistically significant associations between caregiver-child discrepancy in HCT readiness at the full-scale and subscale levels (measured by SDS), PROMIS-A scores (anxiety), and child health services utilization (emergency room visits, hospitalizations, and length of stay) in the past year. Significant associations in these analyses were subsequently included as predictors in the linear regression models.

Linear regression analyses were used to examine the associations between caregiver-child discrepancy in HCT readiness and the child’s demographic variables (i.e., child’s age, sex, age at diagnosis, any comorbid mental disorder), anxiety, and health services utilization. In each model, demographic variables, anxiety (if significantly correlated), and health services utilization (if significantly correlated) were included as predictors, and parent-child discrepancy in HCT (measured by SDS) was included as the outcome variable. Child’s race/ethnicity was not included in the analyses because most of the participants were White (79.9 %), and other racial/ethnic subgroups only consisted of 1.9–8.4 % of the sample, which could bias the regression analysis results.^25^

## 3 Results

Recruitment emails were sent to 938 caregivers of children with chronic conditions who attended the therapeutic camp Victory Junction during the study period. Of the 394 child-caregiver dyads who filled out the Qualtrics survey (response rate 42.0 %), 180 dyads (45.7 %) did not complete the caregiver and/or camper survey and were excluded from the data analysis. The remaining 214 (54.3 %) child-caregiver dyads were included in the present study for analysis. Of the 214 caregivers, 200 (93.5 %) had only one child completing the survey, five had two children completing the survey, and one had four children completing the survey. For the six caregivers with multiple children, their ratings of HCT readiness for each child were matched with the specific child in the statistical analysis. The demographic characteristics of the 214 children and 206 caregivers are shown in Table 1, Table 2, respectively. Table 1 also includes descriptive data on children’s health services utilization.

**Table 1:** Demographic Characteristics and Health Services Utilization of Children (*N* = 214).

	*N* (%) or *M* (*SD*)
**Age**	12.15 (2.61) Range: 7–17
**Age at diagnosis**	3.45 (3.57) Range: 0–13
**Sex**	
Male	102 (47.7 %)
Female	112 (52.3 %)
**Race/ethnicity**	
Non-Hispanic White	171 (79.9 %)
Non-Hispanic Black	18 (8.4 %)
Hispanic	11 (5.1 %)
Asian/Pacific Islander	4 (1.9 %)
Other	10 (4.7 %)
**Primary chronic conditions**	
Neuromuscular disorders	39 (18.2 %)
Genetic disorders	28 (13.1 %)
Neurological disorders	25 (11.7 %)
Diabetes	24 (11.2 %)
Immunological disorders	21 (9.8 %)
Blood disorders	18 (8.4 %)
Kidney diseases	16 (7.5 %)
Heart diseases	15 (7.0 %)
Gastrointestinal disorders	13 (6.1 %)
Others	15 (7.0 %)
**Comorbid mental disorders**^a^	
None	115 (53.7 %)
Attention deficit hyperactivity disorder	58 (27.1 %)
Anxiety disorder	49 (22.9 %)
Mood disorder	19 (8.9 %)
Autism spectrum disorder	5 (2.3 %)
Other	15 (7.0 %)
**Health Services Utilization**	
Past-year emergency room visits (*n* = 211)	0.73 (1.16)
Past-year hospitalizations	0.40 (0.80)
Past-year hospital length of stay (in days; *n* = 213)	1.15 (3.19)

a The percentages added to more than 100 % because some caregivers reported their child having more than one mental disorder.

**Table 2:** Demographic Characteristics of Caregivers (*N* = 206).

	*N* (%)
**Relationship to camper**	
Mother	187 (90.8 %)
Father	10 (4.9 %)
Other (e.g., grandparent, foster parent, etc.)	9 (4.3 %)
**Mother’s education level**	
Some high school or less	4 (1.9 %)
High school diploma	16 (7.8 %)
Some college	40 (19.4 %)
2-year or 4-year college degree	87 (42.3 %)
Graduate or professional school	59 (28.6 %)
**Mother employed? (*****n*****= 204)**	
Yes	146 (71.6 %)
No	58 (28.4 %)
**Father’s education level**	
Some high school or less	10 (4.9 %)
High school diploma	41 (19.9 %)
Some college	45 (21.8 %)
2-year or 4-year college degree	73 (35.4 %)
Graduate or professional school	37 (18.0 %)
**Father employed?**	
Yes	181 (87.9 %)
No	25 (12.1 %)

### 3.1 RQ1: caregiver-child discrepancy in HCT readiness

Paired *t*-test results are presented in Table 3. Using the full score of the STAR_x_ Questionnaire and its sub-domains, no statistically significant differences were found between caregiver’s proxy report and child’s self-report. At the single-item level, statistically significant differences were found in two items: item 3 asking how often the child forgot to take their medicines, and item 5 asking how much the child knows about their illness.

**Table 3:** Discrepancy between Child- and Caregiver-Reported Transition Readiness.

	Caregiver proxy-report *mean* (*SD*)	Child self-report *mean* (*SD*)	Paired-*t* test	
			*t(213)*	*p*
STARx-Total	47.75 (9.24)	46.83 (8.69)	1.08	.28
STARx-DK	16.18 (2.55)	15.72 (2.80)	1.78	.08
STARx-SM	17.71 (4.12)	17.60 (3.66)	0.30	.77
STARx-PC	13.86 (4.16)	13.50 (3.89)	0.92	.36
STARx−1	3.92 (1.03)	3.79 (1.16)	1.20	.23
STARx−2	2.68 (1.24)	2.84 (1.16)	−1.34	.18
**STARx−3**	**2.30 (0.98)**	**3.61 (0.92)**	**−14.45**	*
STARx−4	3.29 (1.25)	3.21 (1.41)	0.67	.50
**STARx−5**	**4.63 (0.61)**	**4.36 (0.91)**	**3.57**	*
STARx−6	4.43 (0.84)	4.27 (0.92)	2.04	.04
STARx−7	4.44 (0.84)	4.27 (0.97)	2.03	.04
STARx−8	4.00 (1.18)	3.93 (1.10)	0.71	.48
STARx−9	3.63 (1.25)	3.65 (1.21)	−0.24	.81
STARx−10	2.94 (1.52)	2.72 (1.54)	1.49	.14
STARx−11	3.64 (1.32)	3.92 (1.18)	−2.18	.03
STARx−12	3.43 (1.41)	3.51 (1.24)	−0.73	.47
STARx−13	3.03 (1.25)	2.77 (1.20)	2.27	0.2

*Note*. STARx-Total: total transition readiness score; STARx-DK: disease knowledge subscale score; STARx-SM: self-management subscale score; STARx-PC: provider communication subscale score.

The Bonferroni-adjusted significance level was.0125 for STARx-DK and STARx-PC, and.01 for STARx-SM.

*p < .001 (two-tailed).

### 3.2 RQ2: associations between caregiver-child discrepancy in HCT readiness and demographic factors, anxiety, and health services utilization

Pearson correlation analyses were conducted to identify statistically significant associations between caregiver-child discrepancy in HCT readiness at the full-scale and subscale levels, PROMIS-A scores, and child health services utilization in the past year. At the full-scale level, the SDS for transition readiness was positively associated with anxiety assessed by PROMIS-A Questionnaire (*r* = 0.27, *p* < .01). At the subscale level, the SDSs for disease knowledge, self-management and provider communication were positively associated with PROMIS-A anxiety (*r* = 0.16, *p* < .05; *r* = 0.20, *p* < .01; *r* = 0.29, *p* < .01, respectively). In addition, the SDS for self-management was also positively associated with inpatient use last year (*r* = 0.16, *p* < .05). Based on the results, anxiety was added as a predictor in linear regression models for transition readiness full-scale SDS, disease knowledge subscale SDS, and provider communication subscale SDS. Anxiety and past-year inpatient use were included as predictors in linear regression models for self-management subscale SDS.

Table 4 presents the linear regression analysis results. For all models, the overall regression was statistically significant (see Table 4), and the adjusted *R*^2^ values ranged from 0.06 to 0.08, indicating a small effect size.^26^ At the STAR_x_ Questionnaire full-scale level, age was negatively associated with the SDS for HCT readiness (β = −.14, *t* = -2.02, *p* < .05), while anxiety was positively associated with the SDS for HCT readiness (β =.23, *t* = 3.18, *p* < .01). At the subscale level, age (β = −.16, *t* = -2.26, *p* < .05) and age at diagnosis (β = −.14, *t* = -2.08, *p* < .05) were negatively associated with the SDS for disease knowledge. Anxiety was positively associated with the SDS for self-management (β =.16, *t* = 2.19, *p* < .05) and the SDS for provider communication (β =.28, *t* = 3.89, *p* < .001).

**Table 4:** Linear Regression Analysis Results.

	Predictor statistics			Model statistics		
	β	*t*	*p*	*F*	*df*	Adjusted *R*^*2*^
**DV 1: STARx-Total SDS**				4.63***	(5, 208)	0.08
**Age**	**−.14**	**−2.02**	*			
Sex (ref: female)	.02	0.35	.73			
Age at diagnosis	−.08	−1.19	.24			
Any mental disorder (ref: no)	.04	0.56	.58			
**Anxiety**	**.23**	**3.18**	**			
**DV 2: STARx-DK SDS**				4.28***	(5, 208)	0.07
**Age**	**−.16**	**−2.26**	*			
Sex (ref: female)	.09	1.32	.19			
**Age at diagnosis**	**−.14**	**−2.08**	*			
Any mental disorder (ref: no)	.12	1.71	.09			
Anxiety	.10	1.38	.17			
**DV 3: STARx-SM SDS**				3.16**	(5, 208)	0.06
Age	−.13	−1.89	.06			
Sex (ref: female)	.02	0.28	.78			
Age at diagnosis	−.06	−0.85	.39			
Any mental disorder (ref: no)	.02	0.34	.73			
**Anxiety**	**.16**	**2.19**	*			
Past-year inpatient use	.12	1.79	.07			
**DV 4: STARx-PC SDS**				3.99**	(6, 207)	0.07
Age	−.06	−0.85	.39			
Sex (ref: female)	−.02	−0.36	.72			
Age at diagnosis	−.01	−0.20	.85			
Any mental disorder (ref: no)	−.04	−0.52	.60			
**Anxiety**	**.28**	**3.89**	***			

*Note*. DV = dependent variable; SDS = standardized difference score; STAR_x_-Total: total transition readiness score; STAR_x_-DK: disease knowledge subscale score; STAR_x_-SM: self-management subscale score; STAR_x_-PC: provider communication subscale score.

* *p* < .05,

** *p* < .01,

*** *p* < .001 (two-tailed).

## 4 Discussion

### 4.1 RQ1: caregiver-child discrepancy in HCT readiness

The current study investigated the discrepancy in child self-reported and caregiver proxy-reported HCT transition readiness, as well as its associations with demographic factors, anxiety, and health services utilization. While no statistically significant discrepancies were identified at the full-scale and subscale levels, the single-item level analysis revealed differences in two items related to the child’s medication forgetfulness and disease knowledge.

On average, children rated higher in the frequency of forgetting to take medications than their parents did in proxy ratings, indicating more frequent self-reported nonadherence. This may reflect awareness gaps between caregivers and children (e.g., caregivers underestimating missed doses if medication-taking occurs privately). Our finding aligns with previous research in children with asthma, where parents often overestimate children’s adherence.^27^, ^28^ However, our finding contrasts with a study in AYAs with Turner syndrome, where caregivers rated children’s medication management lower than children themselves.^9^ The inconsistency may be due to differences in management demands of these conditions that can influence the level of caregiving monitoring and involvement. Meanwhile, it is important to recognize that management needs vary both across and within conditions, with some individuals requiring intensive or lifelong care regardless of diagnosis. Further research could help clarify how management demands may contribute to this variation in caregiver-child reporting accuracy.

On the other hand, our study showed that caregivers rated their child’s understanding of illness more positively than the children did themselves, suggesting that caregivers may feel more confident about their child’s disease knowledge. This may reflect misaligned expectations and communication gaps between caregivers and children (e.g., caregivers assuming comprehension without verification). This finding is inconsistent with previous studies showing that parents rated lower in overall transition readiness than their children with chronic illness.^9^, ^17^ In AYAs from specialty clinics for adolescent medicine, cystic fibrosis, diabetes, and myelodysplasia/spina bifida, there were no statistically significant differences in the overall HCT readiness ratings in caregiver-child dyads.^10^

These mixed results may be partly explained by our linear regression analyses, which found that age was negatively associated with the SDS scores for the STAR_x_ full scale and disease knowledge subscale. Our sample, with an age range of 7–17 years (mean = 12.15), is similar to Bender's two studies: 7–12 years (mean = 10.9 years)^27^ and 8–18 years (mean = 11.175).^28^ On the other hand, it contrasts with the older age ranges in the other studies: 12–25 years (mean = 16) in Patel et al.^9^ and 16–25 years (mean = 19.8) in Sawicki et al.^10^ It is possible that, as children grow older, they become more confident in their disease knowledge and HCT readiness, as they learn more about their illness and take on greater responsibilities in their care management.^8^, ^10^

### 4.2 RQ2: associations between caregiver-child discrepancy in HCT readiness and demographic factors, anxiety, and health services utilization

In addition to age at time of consent, age at diagnosis was also negatively associated with the SDS score for disease knowledge. Specifically, for children diagnosed with chronic conditions at a younger age, caregivers tend to report that their children have greater disease knowledge than the children themselves perceive. Caregivers may assume that, due to their children’s extensive lived experience with the condition from an early age, they possess substantial knowledge about their illness. However, children may not internalize disease-related information to the same depth or detail that caregivers believe, thus feeling less knowledgeable about their disease. With limited literature on this topic, future research could focus on identifying the sources of these discrepancies.

Furthermore, anxiety was positively associated with the SDS scores for the STAR_x_ full scale, self-management subscale, and provider communication subscale. This suggests that higher caregiver ratings of a child’s HCT readiness are associated with greater anxiety in the child. Based on prior research,^12^, ^13^, ^14^, ^15^ we interpret that anxiety may contribute to caregiver-child discrepancies. Specifically, studies suggest that anxiety may hinder children’s ability to develop essential self-management skills and effective engagement in transition-related tasks,^14^, ^15^ leading to lower self-reported HCT readiness.^13^ Meanwhile, caregivers of anxious children may misinterpret anxiety-driven behaviors, such as frequent questioning about health, as indicators of maturity and responsibility rather than distress, resulting in an overestimation of their child’s HCT readiness.

Alternatively, it is also possible that greater discrepancies may lead to heightened anxiety. High caregiver expectations around disease management may place additional pressure on the child, leading to increased stress and anxiety.^29^ In such cases, the mismatch between caregiver and child perceptions could hinder effective collaboration and preparedness during the HCT to adult-focused care and, in turn, increase anxiety.^13^ Finally, other factors may contribute to both anxiety and caregiver-child discrepancies. For instance, family engagement and support could affect the caregiver’s and child’s perceptions of HCT readiness^30^, ^31^ and anxiety.^32^

### 4.3 Study limitations

In this sample, the majority of the children (79.9 %) were non-Hispanic White, and most caregivers were employed (71.6 % mothers, 87.9 % fathers). These characteristics may limit the generalizability of the results to a more diverse population. Future research could specifically examine factors associated with caregiver-child discrepancies in HCT readiness among racially and ethnically minoritized children and families with limited socioeconomic resources.

Another limitation is the reliance on self-reported measures for assessing the child’s HCT readiness, anxiety, and health services utilization. Self-reported data may be influenced by subjective perceptions, potentially introducing inaccuracies in the results. Future research could incorporate objective measures of HCT readiness, such as the TRxANSITION Index,^33^ to evaluate the child’s readiness more accurately. When feasible, retrieving medical records could also provide more reliable health services utilization measures, but in our sample, children and adolescents receive health services in multiple health institutions with different electronic health records systems.

Furthermore, the study’s cross-sectional design limits the ability to make causal inferences about the relationships between anxiety and HCT readiness discrepancies. As discussed earlier, this relationship may be bidirectional and influenced by other factors. Future research could use a longitudinal design to provide valuable insights into the relationship between caregiver-child discrepancies in HCT readiness and anxiety.

Finally, the linear regression models in this study had low adjusted *R*^2^, meaning that the independent variables together only explained a small proportion (6 %-8 %) of the variance of the HCT readiness discrepancies. This suggests that other factors contributing to these discrepancies, such as the child’s healthcare responsibilities and perceived barriers to medical regimens,^34^ were not fully considered. Future research could include these variables to gain a more comprehensive understanding of caregiver-child discrepancies in HCT readiness.

### 4.4 Practice implications

This study demonstrated that children self-reported missing doses more often and reported lower disease knowledge than their caregivers’ proxy reports, suggesting the need to promote medication adherence and disease knowledge in children. This creates an impetus for promoting interventions that target children’s understanding of their chronic illness and practices in medication adherence. Technology-based interventions such as text message reminders and adherence-focused mobile apps have shown promise in improving adherence and warrant further exploration in the literature to improve children’s knowledge of their chronic conditions and medications.^35^, ^36^

Furthermore, the caregiver-child discrepancies highlight the value of involving both caregiver and child perspectives in transition readiness assessments and engaging families in transition planning. It is important for practitioners to address caregiver-child perception gaps through collaborative communication, shared decision-making, and skill-building initiatives. Family-centered care approaches, such as creating and maintaining respectful relationships with children and caregivers, building shared understanding through effective communication, providing flexible and context-sensitive care, and supporting family autonomy and agency,^37^ can help promote alignment in perceptions of transition readiness and improve HCT outcomes.^38^

This study also revealed that higher caregiver proxy ratings of HCT readiness were associated with greater child anxiety, stressing the need for interventions that reduce anxiety to support children with chronic conditions in achieving successful HCT. Incorporating patient education on chronic illness and psychotherapy targeting anxiety management into individualized HCT planning may help reduce children’s anxiety about their condition and the transition process.^39^

Further, this study showed that caregiver ratings tend to be higher in younger children and children diagnosed at a younger age. This suggests a role for early, effective interventions for transition planning to mitigate differences in caregiver-child ratings and associated negative outcomes. For example, one study focusing on younger children demonstrated the acceptability and feasibility of caregiver training sessions teaching behavioral skills for improving child medication adherence in pediatric cancer patients.^40^

## 5 Conclusion

Caregiver-child discrepancies in HCT readiness were found in the areas of disease knowledge and medication adherence, with caregivers rating their child’s HCT readiness more favorably. Greater caregiver proxy ratings were associated with younger child age, earlier age at diagnosis, and elevated child anxiety. These findings suggest the importance of incorporating both caregiver and child perspectives in transition readiness assessments and providing tailored interventions to reduce the caregiver-child perceptual gaps and facilitate the HCT process.

## Funding/Financial Statement

This research did not receive any specific grant from funding agencies in the public, commercial, or not-for-profit sectors.

## Ethical statement

This manuscript adheres to the ethical standards for research involving human participants and received approval from the Institutional Review Board at the University of North Carolina at Chapel Hill (IRB study # 17–1547), where the research team was affiliated during the study period. It has not been published or submitted elsewhere, and the authors have no conflicts of interest to disclose. Additionally, no external funding was received for this work. All authors participated in the intellectual content, conception and design of the paper, took public responsibility for it and have agreed to have their name listed as contributors.

## CRediT authorship contribution statement

**Eugene Maung:** Writing – review & editing, Writing – original draft. **Yunzhen Huang:** Writing – review & editing, Writing – original draft, Visualization, Project administration, Methodology, Investigation, Formal analysis, Data curation, Conceptualization. **Maria Díaz-González de Ferris:** Writing – review & editing, Supervision, Resources. **Chad Coltrane:** Writing – review & editing, Resources. **Stephen R. Hooper:** Writing – review & editing, Formal analysis.

## Declaration of Competing Interest

This research did not receive any specific grant from funding agencies in the public, commercial, or not-for-profit sectors. We have no conflict of interest to disclose.

## Data Availability

Data will be made available on request.

## References

[1] World Health Organization. Adolescent and young adult health. Published November 26, 2024. Accessed July 23, 2025. https://www.who.int/news-room/fact-sheets/detail/adolescents-health-risks-and-solutions.

[2] Lupu M., Ioghen M., Perjoc R. (2023). The importance of implementing a transition strategy for patients with muscular dystrophy: from child to adult—insights from a tertiary centre for rare neurological diseases. Children.

[3] Demirok A., Benninga M.A., Diamanti A. (2024). Transition from pediatric to adult care in patients with chronic intestinal failure on home parenteral nutrition: how to do it right?. Clin Nutr.

[4] Uzark K., Afton K., Yu S., Lowery R., Smith C., Norris M.D. (2019). Transition readiness in adolescents and young adults with heart disease: can we improve quality of life?. J Pedia.

[5] Varty M., Popejoy L.L. (2019). A systematic review of transition readiness in youth with chronic disease. West J Nurs Res.

[6] White P.H., Cooley W.C. (2018). Supporting the health care transition from adolescence to adulthood in the medical home. Pediatrics.

[7] Cooley W.C., Sagerman P.J. (2011). Supporting the health care transition from adolescence to adulthood in the medical home. Pediatrics.

[8] Ferris M., Cohen S., Haberman C. (2015). Self-management and transition readiness assessment: development, reliability, and factor structure of the STARx questionnaire. J Pedia Nurs.

[9] Patel N., Klamer B., Davis S., Nahata L. (2021). Patient-parent perceptions of transition readiness in Turner syndrome and associated factors. Clin Endocrinol (Oxf).

[10] Sawicki G.S., Kelemen S., Weitzman E.R. (2014). Ready, set, stop: mismatch between self-care beliefs, transition readiness skills, and transition planning among adolescents, young adults, and parents. Clin Pedia (Philos).

[11] Hayes D., Edbrooke-Childs J., Town R., Wolpert M., Midgley N. (2019). Barriers and facilitators to shared decision-making in child and youth mental health: exploring young person and parent perspectives using the Theoretical Domains Framework. Couns Psychother Res.

[12] Bitsko R.H., Holbrook J.R., Ghandour R.M. (2018). Epidemiology and impact of health care provider–diagnosed anxiety and depression among US children. J Dev Behav Pedia.

[13] Huang Y., Faldowski R., Burker E., Morrison B., Rak E. (2021). Coping, anxiety, and health care transition readiness in youth with chronic conditions. J Pedia Nurs.

[14] Coyne I., Sheehan A., Heery E., While A.E. (2019). Healthcare transition for adolescents and young adults with long-term conditions: qualitative study of patients, parents, and healthcare professionals’ experiences. J Clin Nurs.

[15] Paine C.W., Stollon N.B., Rabelais E. (2014). Barriers and facilitators to successful transition from pediatric to adult inflammatory bowel disease care from the perspectives of providers. Inflamm Bowel Dis.

[16] Saxby N., Ford K., Beggs S., Battersby M., Lawn S. (2019). Developmentally appropriate supported self-management for children and young people with chronic conditions: a consensus. Patient Educ Couns.

[17] Nazareth M., Hart L., Ferris M., Rak E., Hooper S., Van Tilburg M.A.L. (2017). A parental report of youth transition readiness: the parent STARx Questionnaire (STARx-P) and re-evaluation of the STARx child report. J Pedia Nurs.

[18] Irwin D.E., Stucky B., Langer M.M. (2010). An item response analysis of the pediatric PROMIS anxiety and depressive symptoms scales. Qual Life Res.

[19] Varni J.W., Thissen D., Stucky B.D. (2011). PROMIS® parent proxy report scales: an item response theory analysis of the parent proxy report item banks. Qual Life Res.

[20] I.B.M. Corp. IBM SPSS Statistics for Windows, version 28.0. Armonk, NY: IBM Corp; 2021.

[21] Graham J.W., Cumsille P.E., Shevock A.E., Schinka J.A., Velicer W.F., Weiner I.B. (2013). Handbook of Psychology: Research Methods in Psychology.

[22] Etymologia: Bonferroni correction. *Emerg Infect Dis*. 2015;21(2). doi:10.3201/eid2102.et2102. 10.3201/eid2102.ET2102 PMC4313667 25786274

[23] De Los Reyes A., Kazdin A.E. (2004). Measuring informant discrepancies in clinical child research. Psychol Assess.

[24] Ehrlich K.B., Cassidy J., Dykas M.J. (2011). Reporter discrepancies among parents, adolescents, and peers: adolescent attachment and informant depressive symptoms as explanatory factors. Child Dev.

[25] Hong C., Liu M., Wojdyla D.M., Hickey J., Pencina M., Henao R. (2023). Trans-Balance: reducing demographic disparity for prediction models in the presence of class imbalance. J Biomed Inf.

[26] Emerson R.W. (2023). Regression and effect size. J Vis Impair Blind.

[27] Bender B., Wamboldt F., O’Connor S.L. (2000). Measurement of children’s asthma medication adherence by self report, mother report, canister weight, and Doser CT. Ann Allergy Asthma Immunol.

[28] Bender B.G., Bartlett S.J., Rand C.S., Turner C., Wamboldt F.S., Zhang L. (2007). Impact of interview mode on accuracy of child and parent report of adherence with asthma-controller medication. Pediatrics.

[29] Emerson L.M., Ogielda C., Rowse G. (2018). A systematic review of the role of parents in the development of anxious cognitions in children. J Anxiety Disord.

[30] Kim G., Choi E.K., Kim H.S., Kim H., Kim H.S. (2019). Healthcare transition readiness, family support, and self-management competency in Korean emerging adults with type 1 diabetes mellitus. J Pedia Nurs.

[31] Straus E.J. (2019). Challenges in measuring healthcare transition readiness: taking stock and looking forward. J Pedia Nurs.

[32] Bögels S.M., Brechman-Toussaint M.L. (2006). Family issues in child anxiety: attachment, family functioning, parental rearing and beliefs. Clin Psychol Rev.

[33] Ferris M.E., Harward D.H., Bickford K. (2012). A clinical tool to measure the components of health-care transition from pediatric care to adult care: the UNC TRxANSITION Scale. Ren Fail.

[34] Haarbauer-Krupa J., Alexander N.M., Mee L. (2019). Readiness for transition and health-care satisfaction in adolescents with complex medical conditions. Child Care Health Dev.

[35] Lancaster K., Abuzour A., Khaira M. (2018). The use and effects of electronic health tools for patient self-monitoring and reporting of outcomes following medication use: systematic review. J Med Internet Res.

[36] Kangwal C., Thato R., Ua-Kit N., Visudtibhan A. (2024). Interventions to promote medication adherence among children with epilepsy: an integrative review. J Pedia Nurs.

[37] Ridgway L., Hackworth N., Nicholson J.M., McKenna L. (2020). Working with families: a systematic scoping review of family-centred care in universal, community-based maternal, child, and family health services. J Child Health Care.

[38] Kuhlthau K.A., Bloom S., Van Cleave J. (2011). Evidence for family-centered care for children with special health care needs: a systematic review. Acad Pedia.

[39] Petricone-Westwood D., Jones G., Mutsaers B. (2018). A systematic review of interventions for health anxiety presentations across diverse chronic illnesses. Int J Behav Med.

[40] Bouchard E.G., Epstein L.H., Patel H. (2022). Behavioral parenting skills as a novel target for improving medication adherence in young children: feasibility and acceptability of the CareMeds intervention. Pedia Hematol Oncol.

